# Multiparity induces persistent myocardial structural, functional and transcriptomic remodelling in mice

**DOI:** 10.1038/s41598-025-08248-z

**Published:** 2025-07-07

**Authors:** Ruth R. Magaye, Bing H. Wang, Hongyi R. Liu, Shanae Bailey, Rithy Nuon, Sabrina Sutano, Michael Nyugen, Helen Kiriazis, Kyah Grigolon, Luchen Shan, William W. H. Ho, Fumihiko Takeuchi, Daniel G. Donner, David M. Kaye

**Affiliations:** 1 Heart Failure Research Group, Baker Heart and Diabetes Institute, 75 Commercial Road, Melbourne, VIC 3004 Australia; 2 Monash Alfred Baker Centre for Cardiovascular Research, Monash University, Melbourne, VIC Australia; 3 Biomarker Discovery Laboratory, Baker Heart and Diabetes Institute, Melbourne, VIC Australia; 4 Preclinical Cardiology Research Group, Baker Heart and Diabetes Institute, Melbourne, VIC Australia; 5 Baker Department of Cardiometabolic Health, University of Melbourne, Melbourne, VIC Australia; 6 Domain Bioinformatics, Baker Heart and Diabetes Institute, Melbourne, VIC Australia; 7 Department of Cardiology, Alfred Hospital, Melbourne, VIC Australia

**Keywords:** Multiparity, Hypertrophy, Fibrosis, HFpEF, Cardiac remodelling

## Abstract

Pregnancy poses unique hemodynamic and metabolic demands on the heart. Recent data suggests that a history of multiple pregnancies may be associated with a late-term increased risk of heart failure with preserved ejection fraction (HFpEF), however, the mechanistic basis is unknown. We postulated that the repetitive biological and hemodynamic impact of multiple pregnancies could cause persistent myocardial remodelling providing a substrate for subsequent HFpEF. Multiparous (MP) mice (C57Bl/6J) aged 24 months were compared to age-matched non-parous (NP) mice (n = 8) in a blinded analysis. We evaluated blood pressure, body composition, gross weight and histologic cardiac structure. Cardiac function was assessed by echocardiography together with transcriptomic analysis of the left ventricular myocardium. At 15 months post oestrous cycle disruption, MP mice continued to demonstrate a constellation of myocardial alterations including higher heart to tibial length ratio (*P* < 0.05), body weight (*P* < 0.01), and fat mass (*P* < 0.01) compared to NP mice. MP mice had increased isovolumetric relaxation time (*P* < 0.01), increased end-diastolic (*P* < 0.05) and systolic volumes (*P* < 0.005), and mildly reduced left ventricular ejection fractions (LVEF) (*P* < 0.005) compared to non-parous mice. This was accompanied by marked elevations in mRNA levels of cardiac markers; *Myh6* (*P* < 0.01) and *Nppa* (*P* < 0.01). mRNA levels of *Il18* (*P* < 0.05) and the basal extracellular matrix protein–fibronectin (*P* < 0.05) were also increased, with a significant increase in interstitial fibrosis (*P* < 0.05). Bulk RNA sequencing (RNA Seq) further revealed persistent differential expression of 128 genes with over/under-represented pathways involved in extracellular matrix (ECM) regulation and organic anion/ion transport, respectively. Multiparity alters cardiac gene profile and causes myocardial remodelling that persists into later life, providing a potential foundation for the HF phenotypes such as HFpEF. Further studies are required to investigate the nature of the interaction between pregnancy-associated remodelling and other factors known to be associated with HFpEF development.

**Supplementary Information:**

The online version contains supplementary material available at 10.1038/s41598-025-08248-z.

Heart failure (HF) with preserved ejection fraction (HFpEF) is now the dominant phenotype of HF, and it is projected that its prevalence will continue to rise substantially. Whilst some evidence-based treatments, such as SGLT2 inhibitors, have been recently identified^1,2^ and device-based therapies^3^ are being investigated, the impact of these approaches is limited. The pathogenesis of HFpEF is not well understood and, as such, HFpEF research is internationally acknowledged as a priority^4^. Strategies to prevent the development of HFpEF provide a major opportunity to reduce the HFpEF burden. HFpEF is linked epidemiologically to aging, female sex, hypertension, and obesity providing some insights into target populations^5^.

Women are over-represented among patients presenting with HFpEF. We previously showed that women have a more advanced cardiovascular HFpEF phenotype compared to men^6,7^, and hypothesised that several mechanisms could be responsible for the sex differences in HFpEF distribution. We proposed that obstetric history may be an important contributor to HFpEF severity, and we demonstrated that women with a history of 3 or more pregnancies had greater impairments in multiple physiologic parameters including diastolic reserve and pulmonary vascular resistance^8^.

Our studies demonstrated that elevation of the left ventricular filling pressure, most notably during exercise is a cardinal feature of HFpEF^9^. The cardiovascular mechanics that lead to this feature are complex, however, left ventricular diastolic dysfunction together with vascular stiffening are key components. At the tissue level, fibrosis and inflammation are critical contributors to HFpEF development, and studies in HF patients show that fibrosis is a key contributor to LV diastolic performance^10^. Consistent with this notion, anti-inflammatory interventions including drugs and diets have been shown to mitigate the extent of cardiac remodelling in experimental models^11,12^.

In the current study, we aimed to test the hypothesis that multiparity induces a persistent state of myocardial remodelling that could favour the development of HFpEF. To investigate this, we compared baseline myocardial structure and function in aged, post-menopausal mice who were either multiparous (ex-breeders with > 3 pregnancies) or non-parous (virgin mice) with no other experimental disease interventions. The use of multiparous mice in our study provides a novel model to tease out the differences in basal cardiac molecular and transcriptomics profiles of aged multiparous and virgin mice.

## Results

### Multiparous mice had marked differences in body weight and composition

**Fig. 1:**
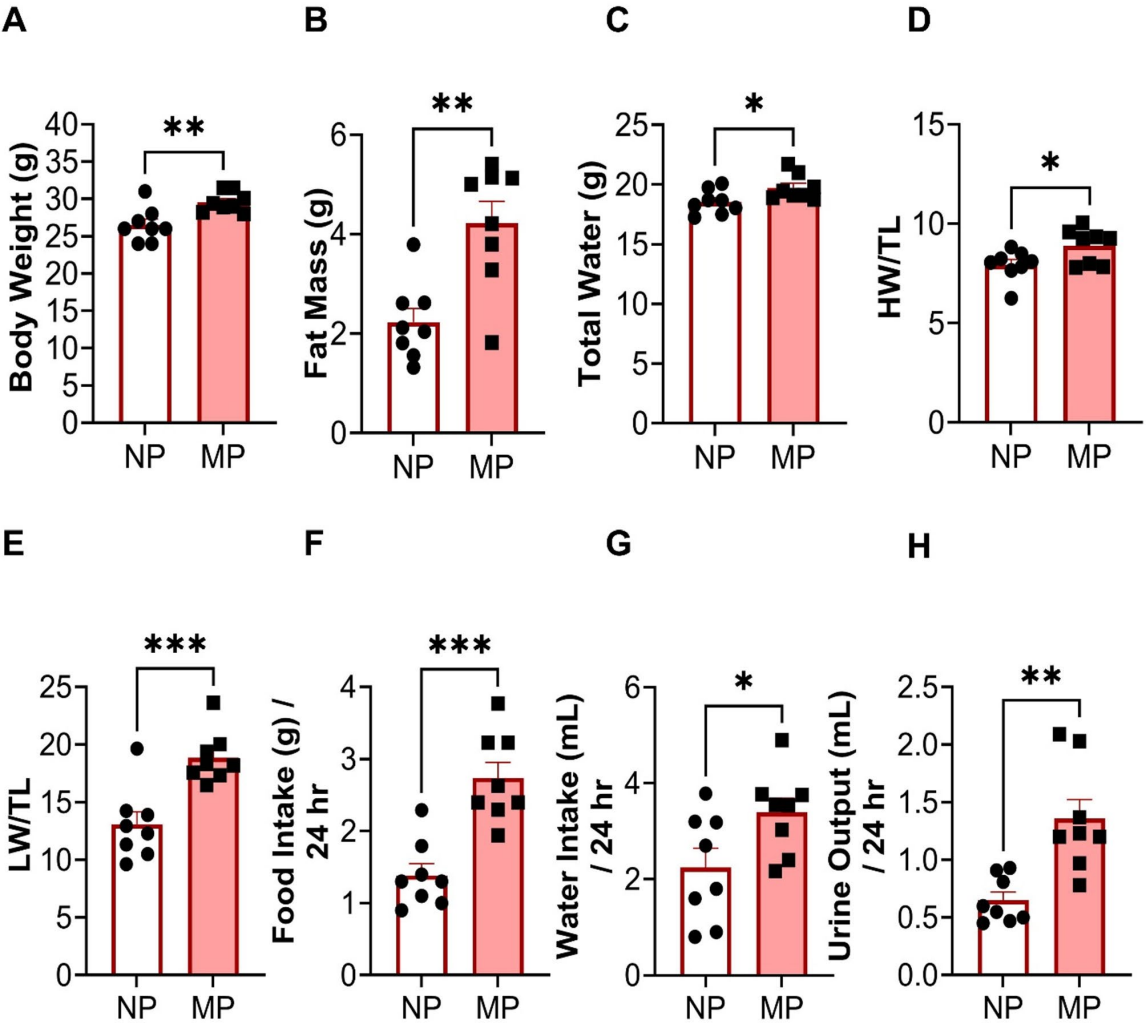
Morphometric, metabolic, and physiological differences between age matched multiparous (MP) and nulliparous (NP) mice. MP mice had increased (**A**) body weight. EchoMRI analysis of body composition showed increased (**B**) fat mass and (**C**) total water (**D**). Quantitative analysis of heart weight-HW and (**E**) lung weight-LW normalised to tibia length-TL. Metabolic caging data showed increased (**F**) food, and (**G**) water intake and (**H**) urine output for MP mice within 24 h. MP, n = 8, NP, n = 8. *P < 0.05, **P < 0.005, ***P < 0.001, MP vs NP. Unpaired two tailed t-test, with Mann–Whitney test for post hoc analysis. Data presented as mean ± standard error of mean (SEM).

MP female mice had significantly increased body weight compared to NP mice (29.5 ± 0.5 vs. 26.5 ± 0.8 g, *P* < 0.005, Fig. 1A). This increase in body weight was coupled with a significant increase in fat mass (4.2 ± 0.4 vs. 2.2 ± 0.3 g, *P* < 0.005) and total water (19.7 ± 0.4 vs. 18.6 ± 0.4 mL, *P* < 0.05, Fig. 1B,C). There were no differences in lean mass and free water between MP and NP mice (Table 1). MP mice also had significantly greater heart (8.9 ± 0.3 vs. 7.9 ± 0.3 mg, *P* < 0.05) and lung (18.9 ± 0.8 vs. 13.1 ± 1.1, *P* < 0.001) weights when normalised to tibia length (Fig. 1D,E). Metabolic caging data showed that MP mice consumed more food (2.7 ± 0.2 vs. 1.4 ± 0.2 g/24 h, *P* < 0.001, Fig. 1F) and water (3.4 ± 0.3 vs. 2.2 ± 0.4 mL/24 h, *P* < 0.05, Fig. 1G) in 24 h than NP mice, which correlates with the increased body weight, fat mass and urine output (1.4 ± 1.2 vs. 0.7 ± 0.1 mL/24 h, *P* < 0.005, Fig. 1H). There were no differences in non-fasted blood glucose levels (Table 1).

**Table 1:** Effects of multiparity on morphometric parameters and metabolism.

Parameters	NP *(n* = *8)*	MP *(n* = *8)*
Heart wt/tibial length, mg/mm	7.94 ± 0.27	8.89 ± 0.31*
Lung wt/ tibial length, mg/mm	13.07 ± 1.1	18.87 ± 0.8***
Kidney wt/tibial length, mg/mm	23.8 ± 0.97	23.16 ± 0.42
Liver wt/tibial length, mg/mm	90.34 ± 3.49	89.72 ± 3.02
Brain wt/tibial length, mg/mm	28.12 ± 0.55	29.18 ± 0.4
Spleen wt/tibial length, mg/mm	8.69 ± 0.56	8.1 ± 1.12
Lean mass, g	21.89 ± 0.46	23.13 ± 0.35
Free water, g	0.19 ± 0.03	0.15 ± 0.03
Blood Glucose, mg/mL	9.99 ± 0.92	10.9 ± 1.67

wt, weight; NP, nulliparous; MP, multiparous. *P < 0.05, & ***P < 0.001 MP vs. NP group. Unpaired two tailed t-test, with Mann–Whitney test for post hoc analysis. Data are presented as mean ± SEM.

### Multiparity elevates systolic blood pressure and alters cardiac function

**Fig. 2:**
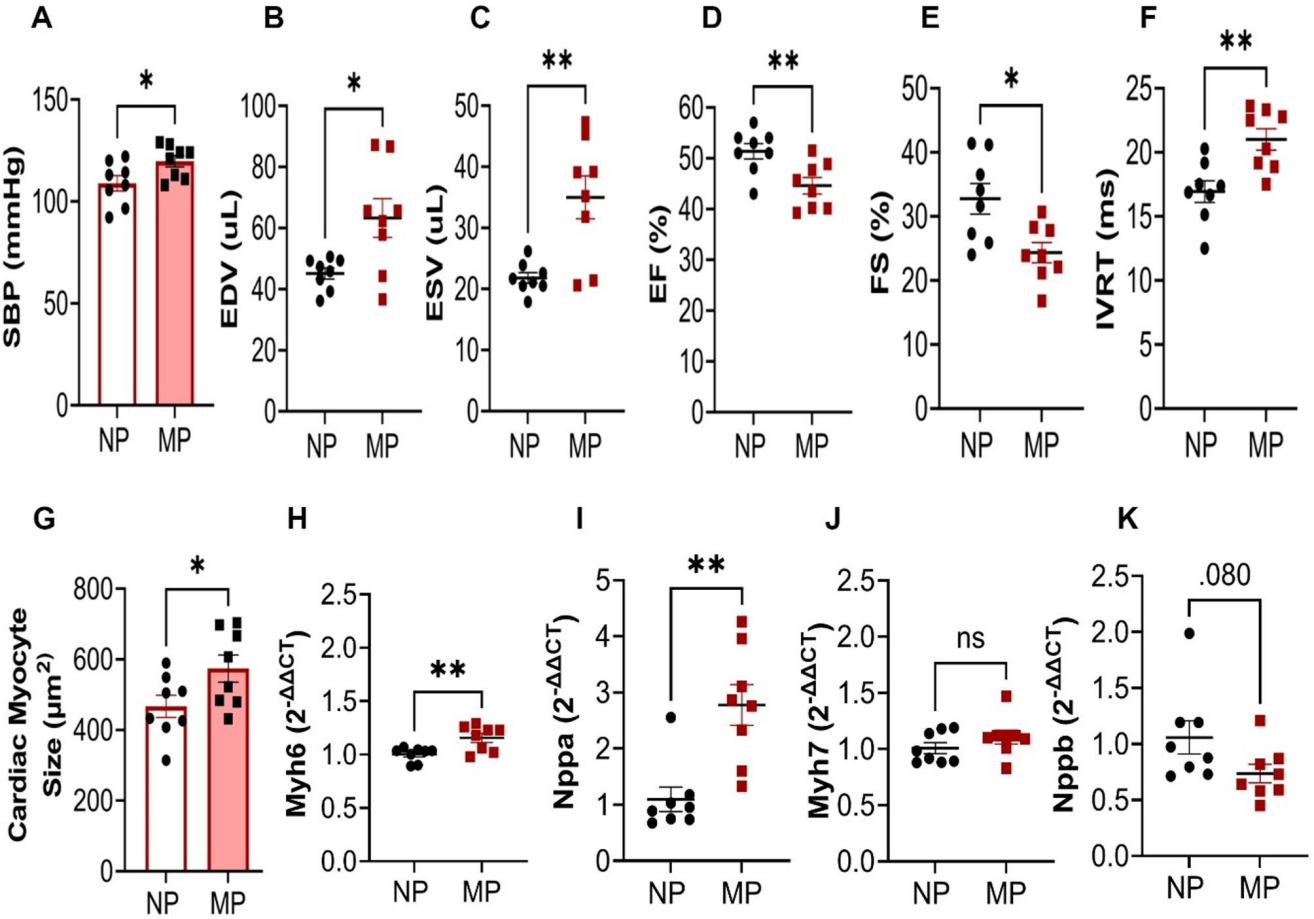
MP mice had elevated systolic blood pressure (SBP), chronic hypertension coupled with myocyte hypertrophy vs. NP mice. (**A**) MP mice had elevated SBP. Echocardiography analysis was suggestive of a chronic hypertension phenotype as indicated by (**B**) increased end diastolic volume- EDV, (**C**) end systolic volume-ESV, (**D**) reduced ejection fraction—EF%, (**E**) and fractional shortening-FS %, and (**F**) prolonged isovolumetric relaxation time- IVRT, in MP mice. (**G**) Myocyte size was increased with increased gene expression of (H) alpha myosin heavy chain- *Myh6*, and (**I**) atrial natriuretic peptide-*Nppa*. There were no significant changes in mRNA expression of (**J**) β myosin light chain- *Myh*7, and (**K**) brain natriuretic peptide- *Nppb* in MP mice. MP, n = 8, NP, n = 8. *P < 0.05, **P < 0.005, ns = *P* > 0.05, MP vs NP. Unpaired two tailed t-test, with Mann–Whitney test for post hoc analysis. Data presented as ± SEM.

Post-menopausal MP mice had significantly higher systolic blood pressure versus age-matched NP mice (119.8 ± 2.8 vs. 109.0 ± 3.7 mmHg *P* < 0.05, Fig. 2A). Multiparity increased both end-diastolic volume (EDV, 63.3 ± 6.3 vs. 45.2 ± 1.8 µL, *P* < 0.05) and end-systolic volume (ESV, 35.0 ± 3.5 vs. 21.8 ± 0.9 µL, *P* < 0.005) with a concurrently lower ejection fraction (44.7 ± 1.6% vs. 51.4 ± 1.5%, *P* < 0.005) and fractional shortening (FS, 24.3 ± 1.5% vs. 32.8 ± 2.3%, *P* < 0.05) (Fig. 2B–E). However, there were no changes in stroke volume (SV) or cardiac output (CO) (Supplementary Table S2). We detected evidence of diastolic dysfunction in MP mice as reflected by a prolongation of the isovolumetric relaxation time (IVRT, 21.0 ± 0.8 vs. 16.9 ± 0.8 ms, *P* < 0.005, Fig. 2F), whilst other measures including the mitral E and A wave velocities, E/A ratio, E/e’ ratio, GLS% or DT were not different in MP mice (Supplementary Table S2).

### Multiparity induces sustained cardiomyocyte hypertrophy

Cardiac myocyte size analysis through H&E staining showed MP mice had substantially greater cardiac myocyte size versus NP mice (574.1 ± 38.2 vs. 466.9 ± 31.3 µm^2^, *P* < 0.05, Fig. 2G). This was accompanied by increased mRNA expression of compensatory markers; alpha myosin heavy chain 6 (*Myh6*, *P* = 0.007) and atrial natriuretic peptide *(Nppa*, *P* = 0.001) (Fig. 2H–J), whilst left ventricular levels of mRNA for the known cardiac stress markers; brain natriuretic peptide (*Nppb*) and beta myosin heavy chain 7 (*Myh7*) were not different (Fig. 2J,K).

### Multiparity has a synergistic effect on cardiac fibrosis, and gene expression of inflammatory markers

**Fig. 3:**
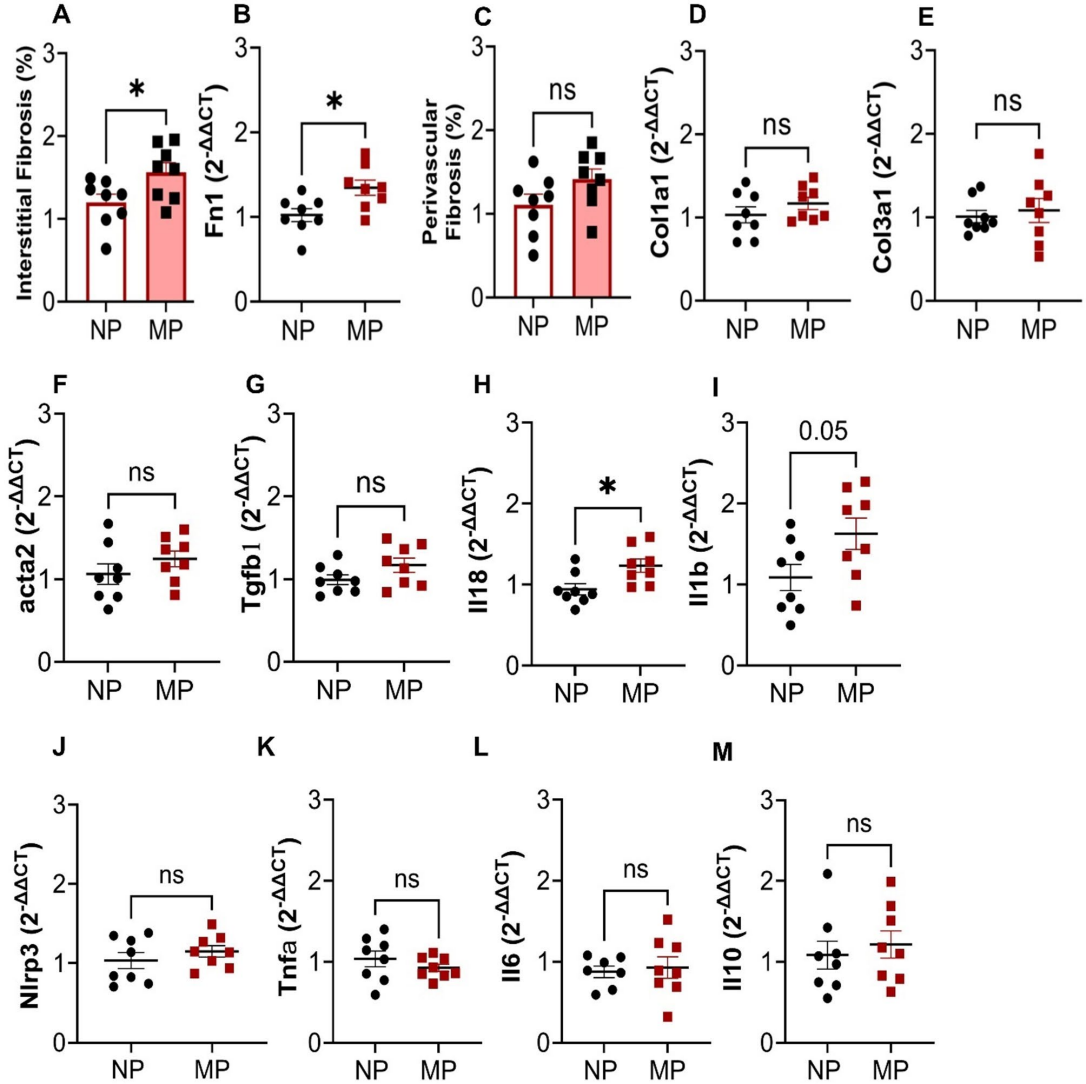
MP mice had increased interstitial fibrosis & basal fibronectin mRNA, and IL-18 mRNA expression. (**A**) Blinded masson’s trichrome staining analysis of X2 sections on Aperio Image Scope showed increased % of interstitial fibrosis in the heart in MP compared to NP mice, (**B**) with no differences in perivascular staining as % of fibrosis to area of vessel wall. (**C**) RT-qPCR analysis showed increased basal fibronectin (*Fn1*), with no alteration in (**D**–**G**) Collagen (Col) 1a1, 3a1, alpha smooth muscle actin (*acta2*) & transforming growth factor β1 (*Tgfb1*) (mRNA expression. The inflammatory marker (**H**) Interleukin 18 (*Il18*) was increased, while there were no significant shift in (**I**) *Il1b* (**J**) Nod like receptor pryin domain containing 3 (*Nlrp3*), (**K**) tumor necrosis factor α (*Tnfa*), (**L**) *Il6* & (**M**) *Il10* mRNA expression in the mid-section of the heart. MP, n = 8, NP, n = 8. *P < 0.05, ns = *P* > 0.05 vs. NP group. Unpaired two tailed t-test, with Mann–Whitney test for post hoc analysis. Data are presented as mean ± SEM.

Multiparity was associated with histological evidence of persistent interstitial fibrosis (1.6 ± 0.1 vs. 1.2 ± 0.1%, *P* < 0.05, Fig. 3A, Supplementary Fig. 1A-B) and increased mRNA expression of the extracellular matrix protein, fibronectin, compared to NP mice (Fig. 3B). The slight increase in fibrosis is also observed in the cardiac sections of the MP mouse hearts, despite both NP and MP hearts showing presence of fibrosis (white arrows) (Supplementary Fig. 1A-B). In contrast it did not appear that perivascular fibrosis (Fig. 3C) was associated with MP mice. Collagen 1a1 (*Col1a1*, Fig. 3D), collagen 3a1 (*Col3a1*, Fig. 3E), alpha smooth muscle actin (*Acta2*, Fig. 3F), and transforming growth factor β1 (*Tgfb1*, Fig. 3G) gene expression levels were not altered. Similarly, regulators of extracellular matrix (ECM) turnover including tissue inhibitors of matrix metalloproteinase 1 and 2 (*Timp1* & *Timp2*) and matrix metalloproteinase 9 and 2 (*Mmp9*, *Mmp2*) were not altered in MP mice (Supplementary Fig. 1C-H). Interestingly, MP mice had considerable elevations in interleukin 18 *(Il18*, *P* = 0.02, Fig. 3H) and interleukin 1β (*Il1b*, *P* = 0.05, Fig. 3I) mRNA expressions, whilst mRNA for other inflammatory mediators including NOD-like receptor family pyrin domain containing 3 (*Nlrp3*, Fig. 3J), tumour necrosis factor α (*Tnfa*, Fig. 3K), interleukin 6 (*IL-6*), and *Il10* mRNA expressions (Fig. 3L,M) were not altered. The numbers of circulating mononuclear cells (Supplementary Table S3) were not different in MP mice.

### Cardiac transcriptomic profile changes in MP mice

**Fig. 4:**
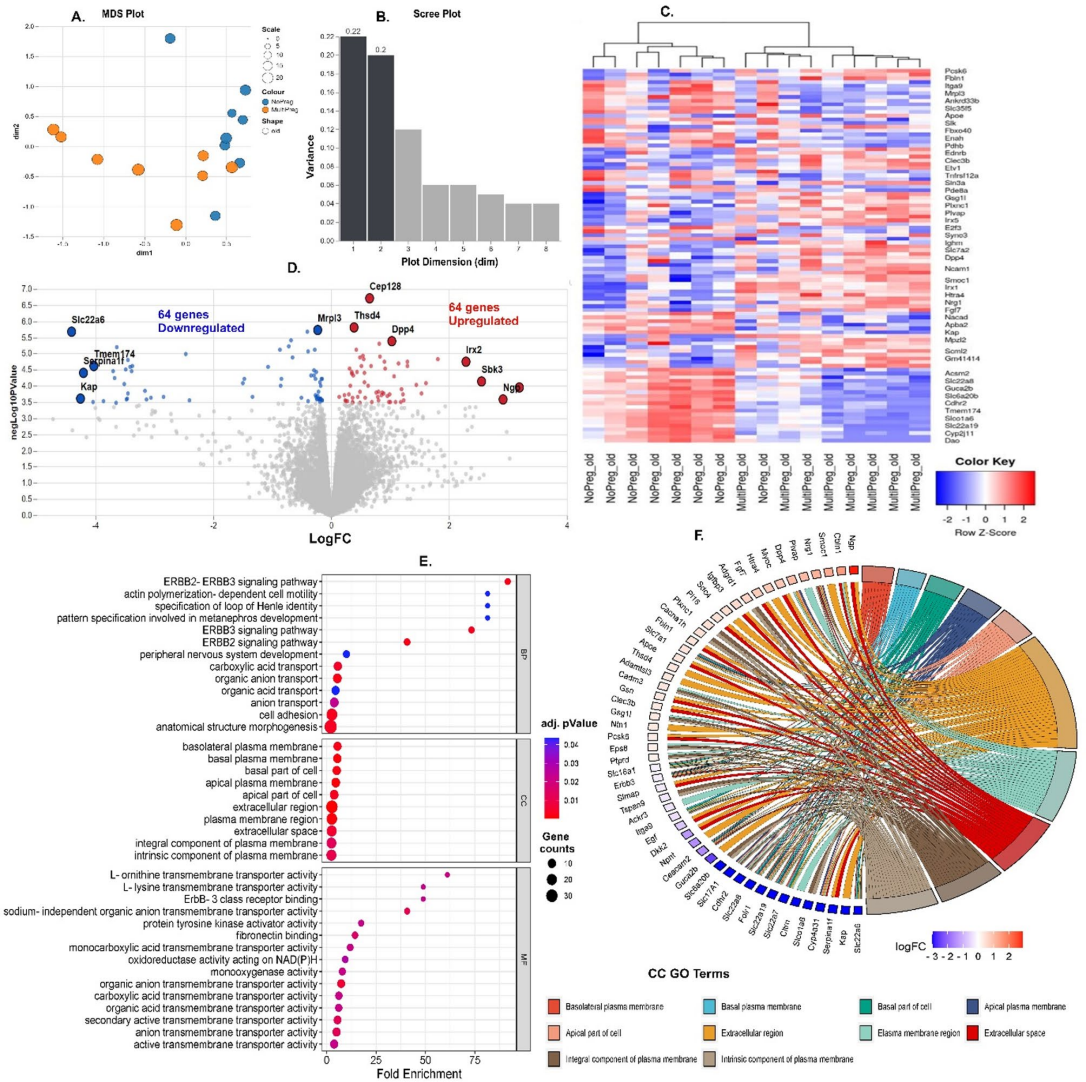
128 genes were differentially expressed in the MP mice compared to NP mice. (**A**,**B**) multi-dimensional (MDS) and scree plot showing 42% of the variance between MP (n = 8) and NP (n = 8) mice were due to dimension 1 and 2. (**C**) Heat map showing log CPM values of the top 100 differentially expressed genes (DEGS) ranked through hierarchical cluster profiling. Only 1 of the NP mice grouped into MP group. (**D**) Volcano plot analysis of DEGs showing 64 upregulated (red) and 64 down regulated genes (blue) in MP mice with *P* < 0.05, and false discovery rate (FDR) < 0.05, at logFC. (**E**) Significantly enriched gene ontology (GO) terms associated with biological processes (BP), cellular components (CC), and molecular function (MF) analysed through ShinyGO 0.8 ranked by FDR and fold change. (**F**) Chord Plot showing of CC GO terms and associated genes and their fold change. Graph generated through SR Plot.

RNA seq analysis to determine the differences in transcriptomic profiles of 24-month-old MP and NP mice revealed 128 differentially expressed genes (DEGs). Approximately 19,000 genes were identified from the RNA seq read set from C57Bl/6 J mouse heart mid-sections. Multi-dimensional scaling analysis (MDS) (Fig. 4A) elucidated that 42% of the variance between MP and NP mice could be attributed to the first two dimensions (Fig. 4B). Unsupervised hierarchical cluster analysis identified 2 distinct groups, with 88% identified as NP and 100% as MP clustering within their respective categories (Fig. 4C). Similarities in cardiac aging effects may have led to the NP mice clustering with the MP mice.

Of the DEGs, half were upregulated and half downregulated (Fig. 4D). Notably, genes such as centrosomal protein 128 (*Cep128*), thrombospondin type 1 domain containing 4 (*Thsd4*), and dipeptidyl peptidase 4 (*Dpp4*) were significantly upregulated (q < 0.01), whereas mitochondrial ribosomal protein L3 (*Mrpl3*), solute carrier family 22 member 6 (*Slc22a6*), transmembrane protein 174 (*Tmem174*), and serine peptidase inhibitor, clade A, member F (*Serpina1f.*) displayed significant downregulations (q < 0.02) in MP mice. c *Slc22a*6, *Tmem174*, *Serpina1f.* including kidney androgen-regulated protein (*Kap*) gene also had a greater fold change in the down-regulated genes. The neutrophil granule protein (*Ngp*), SH3 domain binding kinase family member 3 (*Sbk3*) and Iroquois homeobox 2 (*Irx2*) genes had the higher fold change in the upregulated genes. *Cep128*, *Dpp4* and *Thds4* upregulation were confirmed with real time PCR (Supplementary Fig. 1G-I).

Gene ontology (GO) term analysis through ShinyGO 0.77^13^ revealed a significant enrichment of biological processes and functions in the MP group. Noteworthy pathways included the neuregulin signalling pathways including epidermal growth factor 2–3, Erbb2-3, signalling, (Fig. 4E, and Supplementary Fig. 2A1). Enrichments were also observed in plasma membrane components like the basolateral plasma membrane (Supplementary Fig. 2A2) and in molecular functions including protein tyrosine kinase activity and Erbb3 class receptor binding (Supplementary Figs. 2A3, and 2C). Enriched biological processes in MP mice also encompassed those associated with cardiac contraction (e.g. actin polymerisation-dependent cell motility), kidney development (e.g. specification of the loop of Henley identity), and membrane transport (e.g. carboxylic acid transport;) (Fig. 4E). The extracellular region showed enrichment of genes (Fig. 4F, orange chord) that were also involved in fibronectin binding in the MP mice (Supplementary Fig. 2C, yellow chord), implicating key genes like myocilin (*Myoc*), insulin like growth factor binding protein 3 (*Igfbp3*), syndecan 4 (*Sdc4*), and fibulin 1 (*Fbln1*). *Fbln1* (*P* < 0.001) upregulation in MP mice was confirmed by RT qPCR (Supplementary Fig. 1 J). Additionally, genes associated with membrane transport activities, particularly amino acid (e.g. L ornithine transmembrane transporter) and organic anion/ion transport (e.g. sodium independent organic anion transmembrane transporter activity), were downregulated (Supplementary Fig. 2C).

### Multiparity led to differential expression of genes involved in fibrosis, contraction, and metabolic pathways

**Fig. 5:**
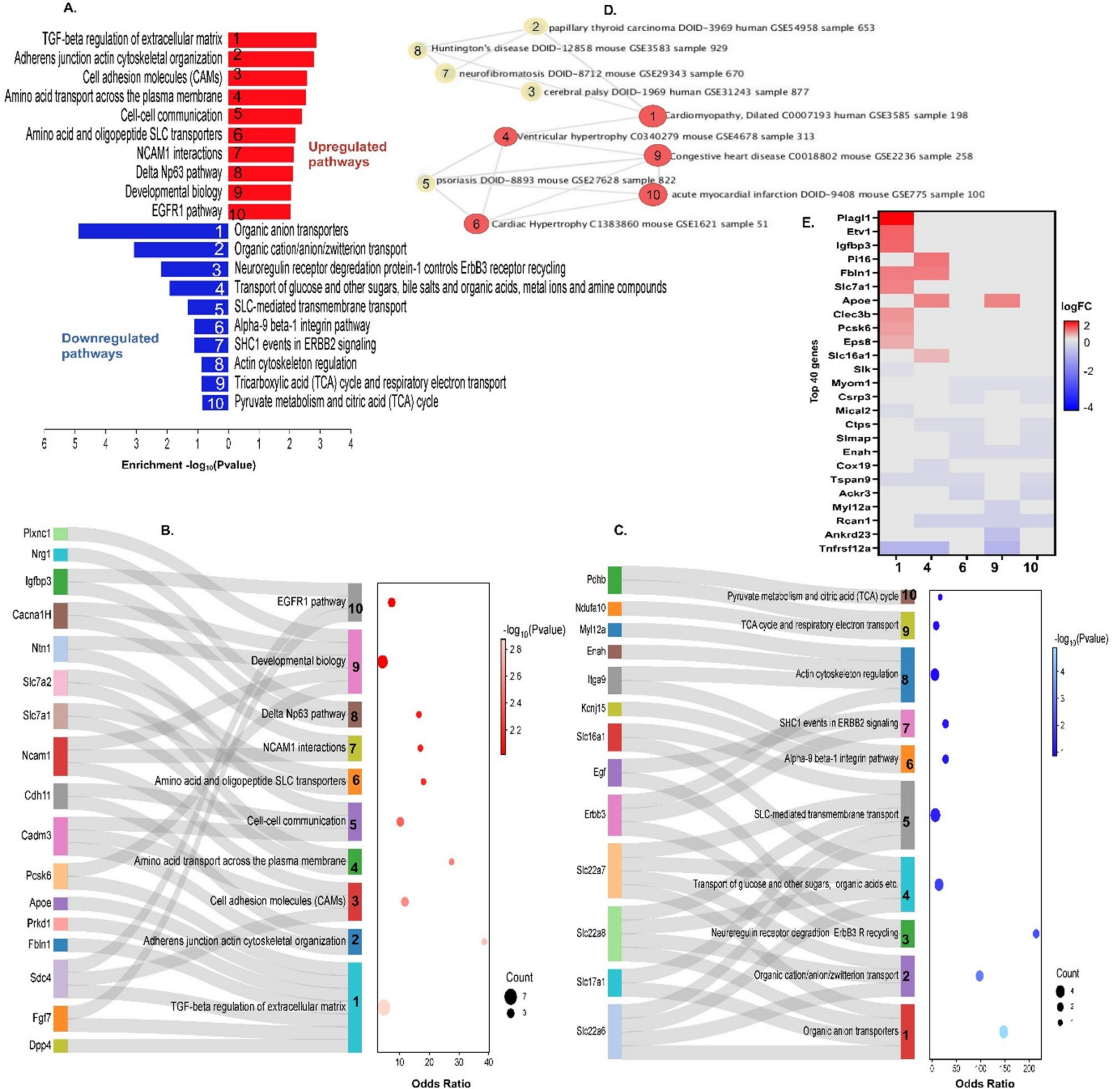
Cardiomyopathy related pathways were enriched by DE genes associated with ECM, cardiac contraction and membrane transport in MP mice. (**A**) Top 10 cellular signalling pathway analysis through Bio-Planet, 2019 for up regulated (red) and down regulated (blue) genes, *P* < 0.05, less stringent FDR of < 0.2. Applying stringent FDR of < 0.05 only produced top 2 pathways. (**B**,**C**) Sankey plot of genes involved in each of the top 10 upregulated and downregulated enriched Bio-Planet pathways within the 64 upregulated and 64 downregulated genes, respectively. (**D**) 5 of the top 10 disease perturbations in GEO UP cardiomyopathy related (red nodes). (**E**) Cluster map of top 40 genes associated with the top 10 disease perturbations, ranked by *P* value, FDR < 0.05. Enrichr was used for these enrichment analysis. Data can be found on https://maayanlab.cloud/Enrichr/enrich?dataset=cebccb1381901ed9014bc6aea9eabb9f.

Biological pathways analysis of the 64 upregulated genes in MP mice through Bioplanet 2019^14^ indicated the TGFβ regulation of the ECM pathway involving genes such as apolipoprotein E (*Apoe*), *Dpp4*, *Fbln1*, fibroblast growth factor 7 (*Fgf7*), protein kinase D1 (*Prkd1*), and *Sdc4* were upregulated. Concurrently, the actin cytoskeleton organisation within adherent junctions was also enhanced in MP mice, evidenced by the upregulation of cell adhesion molecule 3 (*Cadm3*), and cadherin 11 (*Cdh11*) (Fig. 5A,B). In contrast, the pathways governing organic anion transport and actin cytoskeleton regulation were notably downregulated for MP mice and involved several SLC family genes (*Slc22a6*, *Slc17a1*, *Slc22a8*, and *Slc22a7*) and key regulatory genes such as myosin light chain 12A (*Myl12a*), ENAH action regulator (*Enah*), integrin subunit alpha 9 (*Itga9)* and epidermal growth factor (*Egf*) genes. Further pathway analysis revealed suppression of pathways in MP mice critical for mitochondrial metabolism, specifically the tricarboxylic acid (TCA) cycle and respiratory electron transport, implicating genes such as pyruvate dehydrogenase E1 subunit B (*Phdb*) and ubiquinone oxidoreductase subunit A10 (*Ndufa10*) genes (Fig. 5C). These findings suggest preliminary genetic signatures for cardiac fibrosis, hypertrophy, and metabolic insufficiency in the hearts of MP mice.

To explore potential cardiac related disease perturbations within the 128 DEGs, further investigations were conducted using ENRCHR^15,16^ tool against the GEO UP dataset^17,18^. This analysis identified 5 cardiac-related disease perturbations (Fig. 5D). Dilated cardiomyopathy (No. 1 within red node, Fig. 5D) was identified as the most prominent and significant perturbation and involved upregulation of critical genes such as the transcription factor pleomorphic adenoma gene like-1 (*Plagl1*), *Igfbp3*, *Fbln1*, *Slc7a1*, C-type lectin domain family 3, member b (*clec3b*), proprotein convertase subtilisin/kexin type 6 (*Pcsk6*), Egrf pathway substrate 8 (*Eps8*), alongside down-regulation of the multifaceted STE20-like serine/threonine-protein kinase (*Slk*), the microtubule associated monooxygenase, calponin and LIM domain containing 2 (*Mical2*), tetraspanin 9 (*Tspan9*), and TNF receptor superfamily member 12a (*Tnfrsf12a*) (Fig. 5E, x axis column No. 1). Additionally, differential gene expression associated with ventricular hypertrophy, acute myocardial infarction, cardiac hypertrophy, and congestive heart disease were observed in the MP mice (No. 4, 6, 9 & 10 within red nodes, Fig. 5D). Notably, the downregulation of regulator of calcineurin 1 (*Rcan1*), and *Tspan9* were associated with 4 of these cardiac related disease perturbations.

## Discussion

Pregnancy poses unique hemodynamic and metabolic demands on the heart. During pregnancy expansion of the maternal circulating blood volume and cardiac output are associated with adaptive myocardial remodelling which peaks at around 28 weeks of gestation for women^19,20^. Observational clinical studies have suggested that multiparity is associated with hypertension, reduced vascular compliance and LV dysfunction^21,22^. A study conducted in multiparous rats demonstrated reduced vascular compliance and a hypercontractile response to phenylephrine^23^. In a recent study, we demonstrated that women with HFpEF and a history of 3 or more pregnancies had a more exaggerated hemodynamic phenotype^7^.

In the current study, we aimed to further establish whether multiparity establishes a persistent phenotype that may provide a substrate for the development of HFpEF later in life when exposed to other known risk factors such as hypertension. We compared the cardiac structure, function, and transcriptomic profile of multiparous mice at 24 months of age (approximately 69 years old in human years- post-menopausal) vs non-parous age-matched mice. Multiparity in the context of this study is defined as being a retired/ex-breeder, which are known to have > 5 litters before retirement at 10–11 months of age. We specifically investigated the effect of parity per se in the context of aging alone, rather than adding further experimental interventions for potential HFpEF triggers, such as hypertension or obesity, to allow for a focused analysis of parity’s contribution to the pathology. Mice were examined at 24 months of age, representing a substantial period (approximately 15 months) post-oestrous cycle disruption which typically occurs at 10 months in mice.

Our findings for the first time provide evidence for multiparity priming the heart for HF in older females. The distinct cardiac transcriptomic profile and enriched pathway signatures observed in the aged multiparous mice were accompanied by significant phenotypic changes that align with the pathophysiology of HF**.** Echocardiographic investigation demonstrated elements of diastolic dysfunction as exemplified by prolonged isovolumic relaxation time, in the absence of any ongoing stimulus. Left ventricular volumes were increased in the MP mice, potentially of relevance as potential contributors to the pathophysiology of HF with mildly reduced EF (HFmrEF) noting that 25% of HFmrEF patients do transition to HFpEF in females with less severe HF and comorbidities^24^. Transcriptomic analysis identified the downregulation of the myosin light chain regulatory gene, *Myl12a*, which has been associated with prolonged QRS complex duration in genome-wide association studies^25^. Clinical observations support our findings and have shown shifts in EF, and diastolic function in multiparity and twin pregnancies^22,26,27^. This contrasts with singleton pregnancies where EF and contractility rarely change^28^. The reduced EF in our multiparous mice is reflected in older women (66 years and over) who have had ≥ 3 pregnancies^22^, therefore highlighting the novelty of the genomic differences we have shown here. At a histological level, MP hearts were characterised by cardiomyocyte hypertrophy accompanied by an increase in the compensatory cardiac gene marker- *Nppa,* encoding atrial natriuretic peptide (ANP). This increase was not observed in our RNA seq data, perhaps associated with technical differences in qPCR and RNAseq rather than biological. However, RNA seq did reveal the downregulation of genes involved in actin cytoskeleton regulation (*Itga9*, *Enah*, and *Egf*). Both *Itga9* and *Nppa* have been linked to changes in blood pressure^29,30^, while deletion of Enah and downregulation of Egf receptor result in cardiac hypertrophy and contractile dysfunction in mice^31,32^. Plasma ANP levels are known to increase by 40% during pregnancy and up to 148% one week postpartum. This is driven by increased signalling through components of the renin–angiotensin–aldosterone system (RAAS), aimed at regulating BP, peripheral vascular resistance, and hydro-electrolyte balance^33,34^. Accumulating research indicates that an overactive RAAS in aging is a key contributor to heart disease in the elderly^35,36^. Aging-related cellular senescence also contributes to myocyte hypertrophy^37^ which could be exacerbated by multiparity given the significant increase in the atrial natriuretic peptide gene, *Nppa*. Multiparity could also establish an energy-deficient environment indicated by downregulation of *Pdhb*, *Ndufa10*, and the monocarboxylic (pyruvate, lactate etc.) and the carboxylic acid transmembrane transporter genes; *Slc22a19*, *Slco1a6*, *Slc22a6* and *Slc22a8*^38,39^. The organic anion transporters 1 (OAT1) and 3 (OAT3) are encoded by the genes *Slc22a6* and *Slc22a8*. Inhibition of OAT1/3 has been shown to reduce fatty acid oxidation in a chronic heart failure model^40^. However, the parallel reductions in *Pdhb* encoding pyruvate dehydrogenase E1 subunit Beta, which through its multienzyme complex catalyses pyruvate into acetyl-CoA linking glycolysis and the TCA cycle^41^, and *Nduaf10-* an accessory subunit of the mitochondrial respiratory chain complex 1, responsible for its assembly^38^, imply fluctuations in rate and relative oxidation of fatty acids and glucose in the MP mice hearts. It is worth mentioning here the upregulated *Prkd1* and *Erbb3* genes are associated with gestational obesity and have been indicated as important female-specific risk factors for HF^42^. Moreover, it is possible that subtle diet changes could have led to these metabolic changes in the heart given the increased weight of the MP mice. Interestingly, there was a small but significant increase in the *Myh6* gene, encoding alpha myosin heavy chain (αMHC), in the MP mice hearts at 15 months post oestrous cycle disruption. Since these mice do not have experimental interventions, the only distinctive factor is multiple pregnancies, therefore this increase may be a lingering effect of increased cardiac contractility during repeated pregnancies^43^, indicating a compensatory response. Contractility has been observed to increase with raised *Myh6* levels^44^.

In addition to myocardial hypertrophy and altered cardiac energetics, we also detected genetic and phenotypic evidence of interstitial fibrosis in MP mouse hearts. MP mice hearts had significantly increased heart weights. LV wall thickness and mass do increase by 28–52% above pre-pregnancy levels in humans as the hemodynamic load increases indicating the presence of cardiac hypertrophy and remodelling^19,28^. This eventually leads to a temporal increase in mRNA levels of fibrotic markers and increased tissue and perivascular fibrosis^45,46^. Perivascular fibrosis is often noted in animal models where hypertensive stimuli such as L-NAME is employed^47^, therefore it was not observed in the current study which has no added hypertensive stimuli. As the myocardial performance returns to pre-gravid structural function after birth, the myocardium experiences reverse remodelling, which includes a delicate balancing of *Mmp* and *Timp* Transcriptomics of MP mice hearts indicate a state of incomplete reverse remodelling with fibrotic gene signatures in the ECM still differentially expressed at 15 months post oestrous cycle disruption. These signatures included the genes encoding the ECM proliferating proteins such as *Ngp*, *Sdc4*, *Fbln1*, neuregulin 1 (*Nrg1*), and modular calcium binding protein 1 (*Smoc1*). Increased *Nrg1* mRNA expression in the cardiac microvasculature is triggered by neurohormones such as Ang II and mechanical pressure, leading to NRG1 forward signaling through the Nrg1/ErbB pathways affecting cardiomyocyte growth and cardiovascular processes^48,49^. Additionally, through their protein products, *Fbln1* induces ECM deposition, while *Sdc4* and *Smoc1* enhance myocardial stiffness in Ang II-induced and pressure-overloaded heart dysfunction^50–52^.

The matrix metalloproteases Thsd4/ A disintegrin and metalloproteinase with thrombospondin motifs 6 (*Adamst6*) and ADAMST like 3 (*Adamstl3*) were also upregulated and both function in regulating the cardiac ECM^53,54^. However, there were no significant differences in known fibrotic markers including collagens, *Acta2*, Mmps, and Timps genes, in both RNA Seq and qPCR data. A similar Mmp landscape has been noted 7 days post-partum in rats^55^, indicating how quickly they revert to pre-pregnancy levels, thus affirming our findings showing no changes at 15 months post oestrous cycle disruption. Several studies have reported concerted switching of Mmps and Timps before, during, and later in pregnancy^45,46,55,56^. The Mmps act as collagenases and play an important role in the degradation of the ECM^57^. In normal aging, Mmp9 levels are elevated and are involved in the modulation of cytokines, chemokine, hormones, growth factors, and angiogenic factors^58^. Interestingly we observed a significant increase in *Il18* mRNA, which may be a lingering evidence of pregnancy-induced compensatory myocyte hypertrophy in the MP mice^59,60^, which is consistent with the increase in stress induced mRNA expression of ANP.

Despite the lack of change in mRNA levels of *Col1a1*, *Col3a1,* and *Acta2*, we did observe a marked elevation of *Fn1* mRNA indicating an increase in basal collagen levels. Fn1 polymerisation has been shown to be critical in ECM composition, organisation, and stability, and is linked to increased fibrosis in an HF model^61,62^. Fn1 encodes the large basal glycoprotein-fibronectin, which interacts with lysly oxidase to encourage collagen cross linking^63^. This is supported by the RNA seq analysis indicating enrichment of fibronectin binding function with upregulation of *Fbln1*, *Sdc4*, *Igfbp3* and *Nrg1* genes. The activation of Nrg1/Erbb signalling process plays a modulatory role in physiological hemodynamic overload such as pregnancy^48^, and it’s activation months after the last pregnancy maybe linked to epigenetic changes. This signalling pathway is also activated as a compensatory mechanism in response to a failing heart^64^, linked especially to maintaining systolic function and has recently being targeted as therapy for HF^65,66^. Therefore, a reduced Collagen deposition in the aged mice is also gradual and below (1–4% of LV area) the levels seen in pathological states such as a myocardial infarction^67,68^, although the current observations were in the absence of other pro-fibrotic, active stimuli. However, increased cross-linking and reduced degradation due to aging^69^ can result in increase in interstitial fibrosis. The histological features observed in the MP mice in our study are similar to those observed in autopsies of HFpEF hearts which had more interstitial fibrosis than controls with increased hypertrophy^70^. The average age at HFpEF for these patients was 75 years old. Weight gain and increase in fat mass in the MP mice could also have influenced these findings^71^.

Taken together, the findings of this study provide a transcriptomic, functional, and structural basis for clinical observations of an association between multiparity as a potential risk factor for HFpEF. Future studies are required to further establish the nature and extent of a potential interaction between multiparity and other conventional risk factors for HFpEF. From the clinical perspective, our observations may also provide support for closer longitudinal follow-up of multiparous women, particularly in the setting of exposure to other ongoing cardiovascular risk factors.

### Limitations

In the present study, we do not have detailed data regarding the effects of litter size, and number of pregnancies, which could have impacted the results and could account for variations in the clusters for RNA seq data. Therefore, these could have impacted the interpretation of the data provided in this study. Additionally, due to the explorative nature of this study, there is no baseline (ie, at last pregnancy) echocardiography data to compare the echocardiography results at 24 months of age. Additional confirmatory cardiac function assessment such as pressure volume catheterisation and left atria volume index were not performed in this study. We also recommend our findings be interpreted keeping in mind the differences between mice and humans in terms of the number of offspring in a single pregnancy and the gestational periods.

## Materials and methods

### Experimental design for animal studies

All animal experiments were performed in accordance with the National Health and Medical Research Institute Animal Welfare Committee guidelines and the study protocols were approved by the Alfred Medical Research and Education Precinct (AMREP) ethics committee (E/1829/2018/B & E/8252/2022/B). The authors complied with the ARRIVE guidelines. All animals were obtained at 22–23 months of age from Animal Resource Centre, Perth, WA and housed on a 12 h light/dark cycle with food (Irradiated Rat & Mouse Diet-SF00-100, Specialty Feeds, WA) and water provided ad libitum. The MP and NP mice were maintained on SF00-100 normal chow diet consisting of 4.2% fat, 19.6% protein and 59.3% carbohydrates during their aging period and study period. Both MP (n = 8) and NP (n = 8) mice were aged to 24 months of age and were considered post-oestrous cycle disruption by 10 months given that female mice cease reproductive cycles at 16 months^72^. MP mice were selected from the ex-breeding stock whilst NP mice were selected from non-breeding stock. Multiple pregnancies in this study is defined as having > 3 pregnancies determined from first pregnancy at 6–8 weeks, and the last pregnancy before retirement at approximately 40 weeks of age. There were no other experimental interventions conducted on these mice except for the differences in their obstetric history and procedures to measure their morphometric and cardiac differences. Both groups under-went echoMRI testing for body composition analysis, and food and water consumption were assessed through metabolic caging. Both NP and MP mice received normal rodent chow diet. Body weights of all groups were recorded prior to euthanasia. In addition, systolic blood pressure was measured via non-invasive tail cuff plethysmography. Mice were sacrificed by euthanasia using carbon dioxide (CO_2_) exposure, plasma and tissues were taken and stored at − 80 °C until use.

### EchoMRI body composition analysis

EchoMRI was performed on the EchoMRI 4 in 1 body composition analyser (EchoMRI™, TX, USA), following a previously published protocol^73^. Fat mass, lean mass, free water, and total water were determined during the scan which lasted approximately 1 min.

### Metabolic caging

24 h metabolic caging was performed using single mouse metabolic cages (Techniplast, MI, Italy). Mice were housed on a 12 h light/dark cycle with food and water provided ad libitum*.* Body weight, food weight, and water weight and urine volume were recorded over the 24 h period of metabolic caging for quantitative analysis of differences in energy and fluid homeostasis.

### Tail cuff plethysmography for systolic blood pressure measurement

Briefly, mouse was placed in the restrainer, the tail threaded through the tail cuff, and stabilised with the sensory assembly unit on the MC4000 non-invasive multi-channel blood pressure analysis system (Hatteras Instruments, NC, USA). The platform temperature was acclimatised and monitored at 37 °C prior to restraining the mice. 5 preliminary readings were taken before the 10 actual reading cycles by inflating the tail cuff. Pressure readings were taken every minute for 10 min.

### Echocardiography analysis

To assess cardiac function, echocardiography was performed on mice under anaesthesia (Ketamine, Xylazine, Atropine (KXA), 80/8/0.96 mg/kg, i.p.). Echocardiography was performed using a Vevo 2100 (Fujifilm Visualsonics Inc., ON, Canada) echocardiography system with a 40 MHz MS55OD transducer, with mice imaged on an ECG-, temperature-, and respiration-recording platform. Parasternal long axis imaging of the left ventricle was obtained by 2D B-mode and analysed using the VevoLab VevoStrain analysis package to measure left ventricular volumes, ejection fraction (EF) and global longitudinal strain (GLS %). Trans-mitral flow velocity was assessed using Doppler where the early phase (E wave) and late phase (A wave) were examined to determine E/A ratio and deceleration time (DT) was measured from the peak E wave to it’s projected baseline. Similarly, tissue Doppler was performed to assess early diastolic mitral annular velocity (e’), and the ratio E/e’. All traces were analysed in a blinded fashion using the VevoLab analysis software-VisualSonics.

### Quantitative real-time PCR

To assess changes in gene expression, total RNA was isolated from left ventricular tissue using RNeasy Mini Kit (Qiagen, NRW, Germany) following manufacturer instructions. RNA samples were reverse-transcribed into cDNA using High-Capacity cDNA Reverse Transcription Kit on the Veriti Dx 96-well Thermal Cycler (Applied Biosystems, MA, USA). Reverse transcribed cDNA from each sample were plated on a 384-well plate and run for PCR amplification with specific primer pairs for genes of interest (Sigma-Aldrich, MA, USA; Supplementary Table S1). Real-time PCR was performed using Fast SYBR Green Master on the QuantStudio 7 Flex Real-Time PCR System (Applied Biosystems, MA, USA) to determine mRNA levels of the target genes. Gene expression was calculated using comparative CT (ΔΔCT) method normalised to the housekeeper gene, Glyceraldehyde 3-phosphate dehydrogenase (GAPDH).

### RNA sequencing and bioinformatics analysis

To complement the targeted qPCR studies, RNA seq was also performed on mid sections of the left ventricular tissue. Total RNA was extracted, and cDNA was generated using Illumina Stranded Total RNA Prep kit with Ribo-Zero Plus depletion. Sequencing was completed on a NovaSeq X with 150 paired-end reads and sequenced to a depth of a minimum of 50M reads per sample.

#### Data quality control and preprocessing

Upon sequencing, raw reads were demultiplexed by bcl2fastq version 2.20 (RRID:SCR_015058) and fastq files resulted were subjected to preprocessing by TrimGalore suite v0.6.10 (RRID:SCR_011847), involving Illumina adapter and priming site removal using Cutadapt version 4.8 (DOI:10@@@.14806/ej.17.1.200), followed by quality control assessments with FastQC v0.12.1 (http://www.bioinformatics.babraham.ac.uk/projects/fastqc). Before further demultiplexing the splits of fatsq files were concatenated back into 23 samples for further data processing, and Multi-QC version 1.21^74^ was performed to validate the sequencing-quality and -consistency for each split.

#### Alignment and quantification

The filtered reads were mapped to the mouse reference genome (GRCm38.p6.vM25) using STAR aligner version 2.7.11b^75^. BAM files were processed and indexed using Samtools version 1.19.2^76^ and Bamtools version 2.5.2^77^ to provide mapping statistics. HTSeq v0.11.1^78^ was employed for constructing counting matrices, and an aggregated quality control summary was compiled with Multi-QC.

#### Differential expression analysis

Count matrices were processed using base-R version 4.2.2 (https://www.R-project.org) for metadata integration, gene annotation, filtering of lowly expressed genes. After Trimmed Mean of M-values (TMM) normalization by EdgeR version 3.40.2^79^, procedures including mean–variance dependence elimination, empirical Bayes moderation, and quality-weighted VOOM transformation were implemented using limma package version 3.54.2^80^. Glimma version: 2.8.0^81^ was applied for unsupervised sample clustering and other data visualization for the differential gene expression analyses.

#### Downstream analyses

Differentially expressed genes (DEGs) were identified using predefined criteria of adjusted *P*-values (< 0.05). Enrichment and Gene Set Enrichment Analysis (GSEA) of DEGs in context of Gene Ontology (GO)^82^ or KEGG pathway annotations were performed using clusterProfiler version 4.10.1 ^83,84^, Enrichr (https://maayanlab.cloud/Enrichr/#libraries) ^15,16,85^ for Bioplanet 2019 ^14^ and Disease Perturbations in GEO UP ^17,18^ and Pathview version 3.18 ^86^, ShinyGO 0.77 (http://bioinformatics.sdstate.edu/go/) ^13^ and visualised using SR Plot^87^. All enrichment analysis files are supplied in Supplementary Excel Files S4.

### Histological assessment of collagen staining and myocyte size

To assess collagen deposition, 4 mm thick whole heart tissue sections (excluding atria) were subjected to Masson’s Trichrome staining. This was performed by the Monash Histology Platform, Clayton, VIC, Australia. Scanned bright field images from Aperio Slide Scanner using the Olympus FSX100 bright field microscope at X 20 magnification. Analysis was conducted using Aperio Image Scope software version 12.4.6 (Leica Biosystems, HE, Germany). 2 cardiac tissue cross sections per heart were annotated and blood vessels and cavities were excluded before analysing for positive pixel intensity of aniline blue stained collagen fibres. Similarly, to assess cardiomyocyte width, cardiac tissue sections were processed and stained with Hematoxylin & Eosin (H&E). The size of 20 cardiomyocytes/section randomly selected within the left ventricle of 2 cardiac cross sections were analysed by two blinded scientists from slide scanned images.

### Data and statistical analysis

All data analysis were conducted in a blinded manner, with analyst blinded to group allocation for echocardiography, PCR, histology, and morphometrics data analysis. Sample size estimates were based on our prior studies investigating the effects of angiotensin II as a stimulus for cardiac remodelling^11^. Statistical significance was determined using a 2-tailed Student’s t-test, where normality was tested using Shapiro–Wilk test. Mann–Whitney test was used for post hoc analysis. A *P*-value of less than 0.05 was deemed statistically significant, exclusive of RNA seq data which were deemed statistically significant at an adjusted *P*-value of < 0.05 after correcting for multiple testing. All data are expressed as mean ± standard error of the mean (SEM), unless otherwise noted, and statistical analysis was carried out using GraphPad Prism 10.0 software for all other data analysis, while R (version 4.2.2) was used for RNA seq data analysis.

## Electronic supplementary material

Below is the link to the electronic supplementary material.

Supplementary Material 1

Supplementary Material 2

## Data Availability

All data supporting the findings of this study are available upon request. RNA sequencing dataset generated during the current study are available in the Gene Expression Omnibus (GEO) repository and can be accessed here https://urldefense.com/v3/https://www.ncbi.nlm.nih.gov/geo/query/acc.cgi?acc=GSE289, with the token- ircdaaauhnwzpyz. Contact Dr. Bing Wang to access this data if needed.

## Abbreviations

DEGs: Differentially expressed genes
HF: Heart failure
HFpEF: Heart failure with preserved ejection fraction
MP: Multi-parous/multiple pregnancies
NP: No pregnancy (virgin)
RNA Seq: RNA sequencing
