# Supplementary material for: Multiparity induces persistent myocardial structural, functional and transcriptomic remodelling in mice
Source: Sci Rep. 2025 Jul 7;15:24254. doi: 10.1038/s41598-025-08248-z (PMC12234663; doi:10.1038/s41598-025-08248-z)
Supplement: Supplementary file 1 — Supplementary Material 1 [file 41598_2025_8248_MOESM1_ESM.pdf]

SUPPLEMENTARY TABLE

Supplementary Table S1. PCR primer pairs list.

Primer Name	Forward Sequence	Reverse Sequence
Acta2	CATCTTTCATTGGGATGGAG	TTAGCATAGAGATCCTTCCTG
Nppa	GAGAGAAAGAAACCAGAGTG	GTCTAGCAGGTTCTTGAAATC
Nppb	AATTCAAGATGCAGAAGCTG	GAATTTTGAGGTCTCTGCTG
Col1a1	GATTGAGAACATCCGCAGCC	TACTCTCCGCTCTTCCAGTCA
Col3a1	ACTCAAGAGTGGAGAATACTG	AACATGTTTCTTCTCTGCAC
Fn1	CCTATAGGATTGGAGACACG	GTTGGTAAATAGCTGTTCGG
Gapdh	TGAAGCAGGCATCTGAGGG	CGAAGGTGGAAGAGTGGGAG
Il1b	TGCCACCTTTTGACAGTGATG	ATGTGCTGCTGCGAGATTTG
Il6	AAGAAATGATGGATGCTACC	GAGTTTCTGTATCTCTCTGAAG
Il10	CAGGACTTTAAGGGTTACTTG	ATTTTCACAGGGGAGAAATC
Il18	AAATGGAGACCTGGAATCAG	CCTCTTACTTCACTGTCTTTG
Mmp2	TCACTTTCCTGGGCAACAAGT	GCCACGAATAGGCTATATCC
Mmp9	CTTCCAGTACCAAGACAAAG	ACTTGTTCACCTCATTTTG
Nlrp3	GATGCTGGAATTAGACAACTG	GTACATTICACCCAACTGTAG
Tgfb1	GGATACCAACTATTGCTTCAG	TGTCCAGGCTCCAAATATAG
Tnfa	CTATGTCTCAGCCTCTTCTC	CATTGGGAACTTCTCATCC
Timp1	CTAGAGACACACCAGAGATAC	CCCATGAATTTAGCCCTTATG

Timp2	GGATTCAGTATGAGATCAAGC	GCCTTTCCTGCAATTAGATAC
Myh6	AATCCTAATGCAAACAAGGG	CAGAAGGTAGGTCTCTATGTC
Myh7	GTGCCAAGGGCCTGAATGA	TGCTTCCACCTAAAGGGCTG

Acta2- Smooth Muscle Alpha Actin, *Co1a1*-Collagen 1a1, *Col3a1*-Collagen 3a1, *Fn1*-Fibronectin, *Gapdh*- Glyceraldehyde 3-phospate dehydrogenase, *Il1b*- Interleukin-1β, *Il6*-Interleukin 6, *Il10*- Interleukin 10, *Il18*- Interleukin 18, *Mmp2*- Matrix Metalloproteinase 2, *Mmp9*-Matrix Metalloproteinase 9, *Nlrp3*- NOD-like receptor family pyrin domain containing 3, *Nppa*- Atria Natriuretic Peptide, *Nppb*- Brain Natriuretic Peptide, *Tgfb*-Transforming Growth Factor β, *Tnfa*-Tumor Necrosis Factor α, *Timp1*-Tissue Inhibitor of Matrix Metalloproteinase 1, *Timp2*- Tissue Inhibitor of Matrix Metalloproteinase 2, *Myh6*-Myosin Heavy Chain α, *Myh7*- Myosin Heavy Chain β.

Supplementary Table S2. Echocardiography parameters.

**Cardiac Function Parameters**	**NP** ***(n=8)***	**MP** ***(n=8)***
HR, bpm	459±15	458±8
EF, %	51.4±1.5	44.6±1.6 **
GLS, %	12.4±0.8	11.5±0.7
SV, μL	23.4±1.3	28.3±3.1
CO, μL	10.6±0.5	12.4±1.3
E, mm/s	585.5±25.5	632.6±49.2
A, mm/s	299.1±16.5	353.8±36.8
E/A ratio	1.9±0.1	1.8±0.1
e’, mm/s	-19.7±2.0	-20.0±1.8
E/e’	-32.5±4.2	-33.6±1.9
DT, ms	21.1±2	20.6±1.8

HR, heart rate; SV, stroke volume; CO, cardiac output; E, early filling; A, late filling; e’, tissue Doppler early diastolic mitral annular velocity; GLS, global longitudinal strain; DT, deceleration time; NP, non-parous; MP, multiparous. **P<0.01vs. NP group. Unpaired two tailed t-test, with Mann-Whitney test for post hoc analysis. Data are presented as mean ±SEM.

Supplementary Table S3. Whole blood count measurements.

Parameters	NP *(n=8)*	MP *(n=8)*
WBC, 10^3 cells/ μL	748.1±189.9	876.6±318.6
Monocytes, 10^3 cells/ μL	15.0±2.4	18.3±2.2
Lymphocytes, 10^3 cells/ μL	277.6±10.4	453.9±86.6
Neutrophils, 10^3 cells/ μL	26.5±8.2	57.1±23.2
Basophils, 10^3 cells/ μL	70.6±18	71.0±6.1
Platelets, 10^3 cells/ μL	1378.0±230.7	1140.0±213.6
RBC, 10^6 cells/ μL	748.1±189.9	876.6±318.6
HCT, %	422.8±21.0	420.6±17.9
MCV, fL	518.1±27.3	479.6±11.6

HCT, hematocrit; MCV, Mean corpuscular volume; RBC, red blood cells; WBC, White blood cells; NP, nulliparous; MP, multiparous. Unpaired two tailed t-test, with Mann-Whitney test for post hoc analysis. All not statistically significant. Data are presented as mean ±SEM.

SUPPLEMENTARY FIGURE

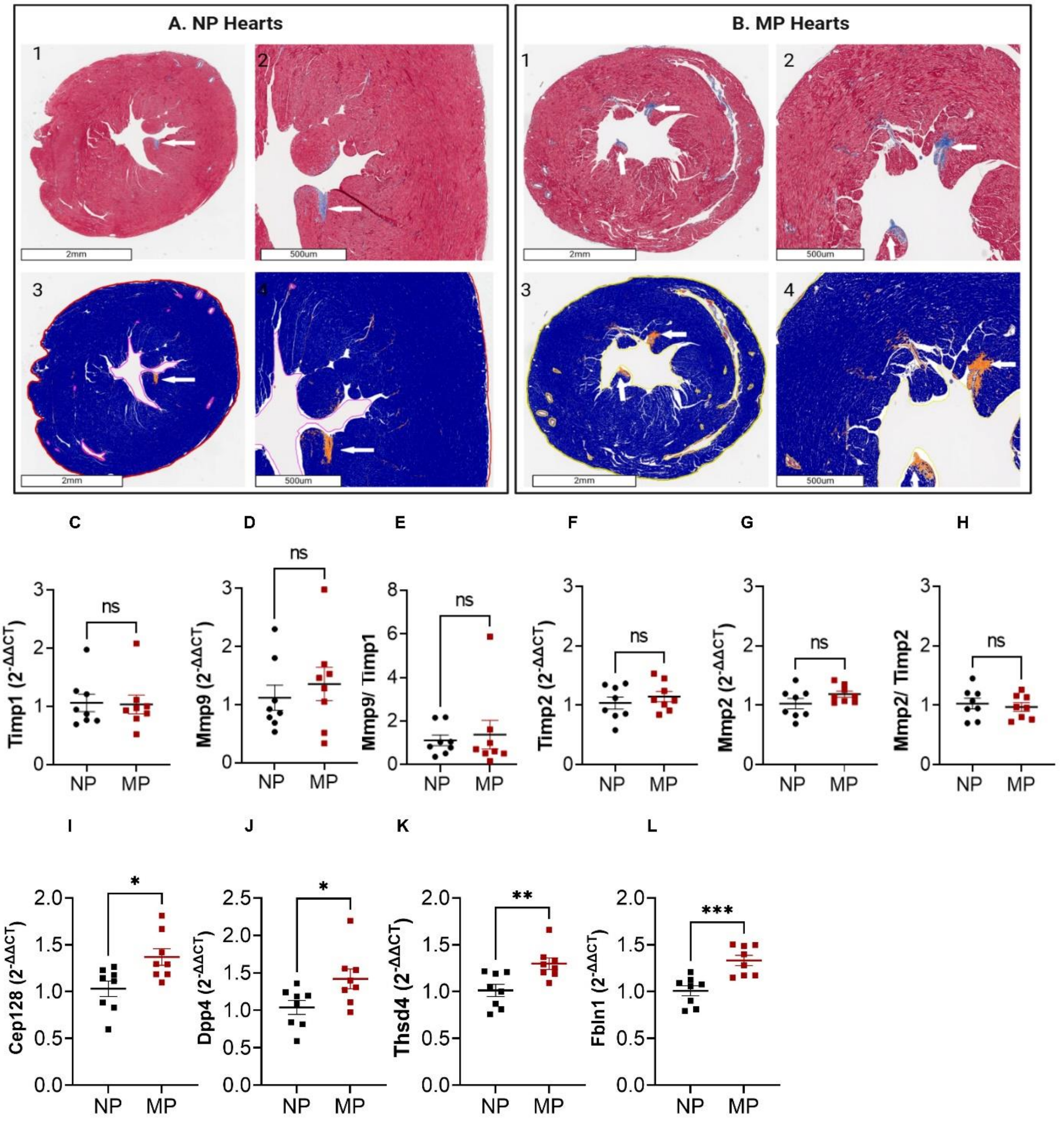

Supplementary Figure 1. There were no differences in extracellular matrix synthesis and degradation gene markers. **A)** Nulliparous and **B)** multiparous mouse hearts stained with mason's trichrome stain (A1-2 and B1-2), and their positive pixel transposed images (A3-4 and B3-4) captured after analysis on Aperio ImageScope software V12 (white arrows show collagen staining). The mRNA levels of **B)** tissue inhibitor of matrix metalloproteinases 1 (*Timp1*), **C)** matrix metalloproteinase 9 (*Mmp9*) and **D)** their ratios were not different between the groups. **E)** *Timp2*, **F)** *Mmp2* and **G)** their ratios were also not significantly different. *Cep128*,

Dpp4, *Thds4* and *Fbln1* mRNA were also significantly increased in RT qPCR (**I-L**). MP, n=8, NP, n=8. ns= *P*<0.05, MP vs. NP group. Unpaired two tailed t-test, with Mann-Whitney test for post hoc analysis. Data are presented as mean ±SEM.

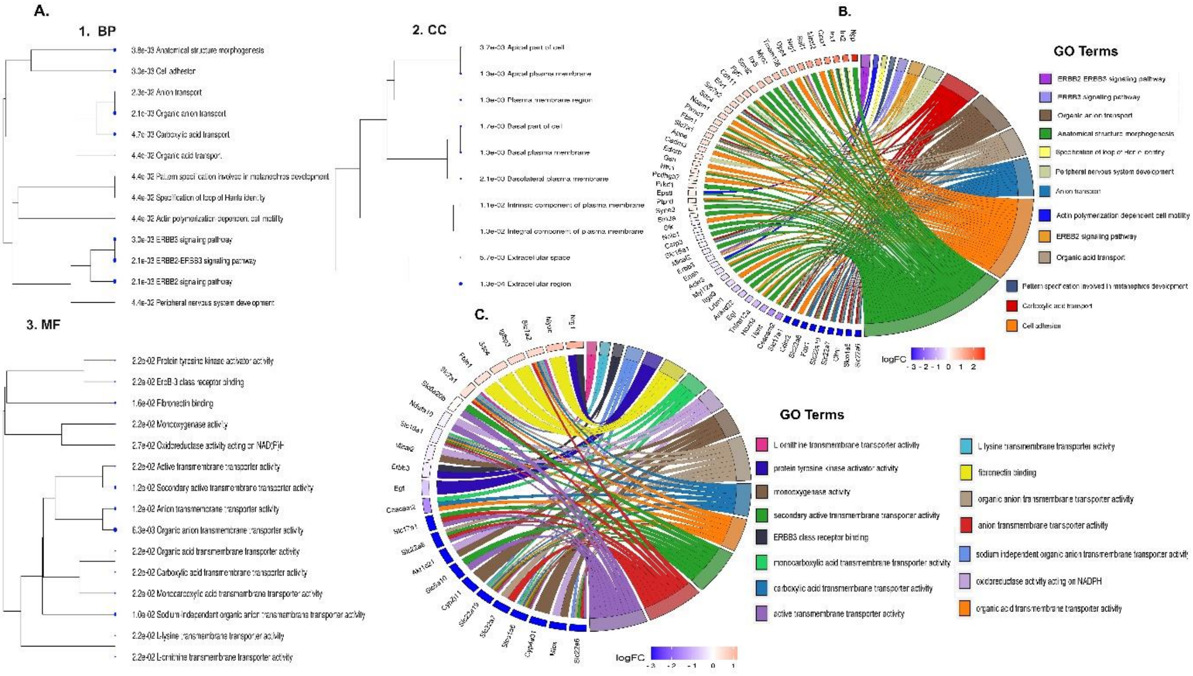

Supplementary Figure 2. Gene ontology (GO) terms enriched by differentially expressed genes in MP (n=8) vs. NP (n=8) mice. Dendrogram of enriched GO terms associated with **A1)** biological processes (BP), **A2)** cellular components (CC), and **A3)** molecular functions (MF) analysed and derived through ShinyGO 0.8, ranked by FDR (<0.05) and fold enrichment. **B)** Chord plot showing high number of genes associated with anatomical structure morphogenesis and cell adhesion process GO terms. **C)** Chord plot showing the GO term fibronectin binding (yellow) molecular function was significantly enriched while genes associated with membrane transport GO terms were highly downregulated. Chord plots generated through SR Plot.
